# Supplementary material for: The SATB1‐MIR22‐GBA axis mediates glucocerebroside accumulation inducing a cellular senescence‐like phenotype in dopaminergic neurons
Source: Aging Cell. 2024 Feb 1;23(4):e14077. doi: 10.1111/acel.14077 (PMC11019121; doi:10.1111/acel.14077)
Supplement: Supplementary file 1 — Appendix S1. [file ACEL-23-e14077-s001.docx]

***SUPPLEMENTAL FIGURES***

*
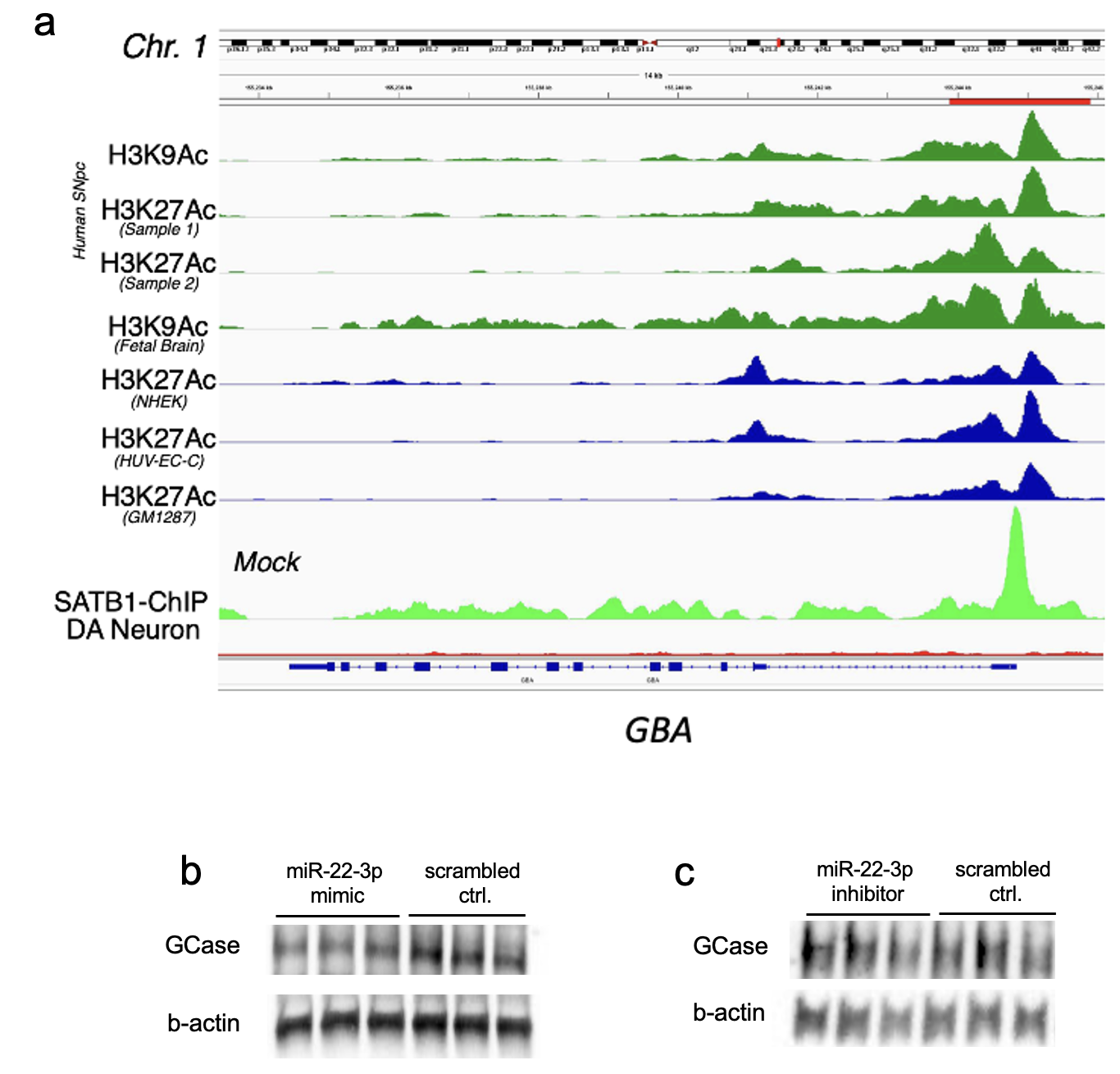
*

**Figure S1. SATB1 binds to the regulatory region of GBA in dopaminergic (DA) neurons and miR-22-3p regulates GCase levels in SK-N-MC cells.**

**a,** ChIP-seq analysis reveals significant binding of SATB1 to the GBA gene in 60-day-old human embryonic stem cell–derived DA neurons. The GBA gene is overlaid with H3K27Ac and H3K9Ac enrichment tracks from human substantia nigra samples, human fetal brain, and three human cell lines. H3K27Ac and H3K9Ac data were obtained from http://www.roadmapepigenomics.com. The chromosomal locus and exons of GBA are shown, with the regulatory region of GBA highlighted by a red line. ChIP-seq was performed in quadruplicate (n=4). **b** and **c,** representative Western blots of GCase from wildtype (b) and SATB1-KO (c) SK-N-MC cells after treatment with miRNA-22-3p mimic and inhibitor, respectively (n=3, each).

*
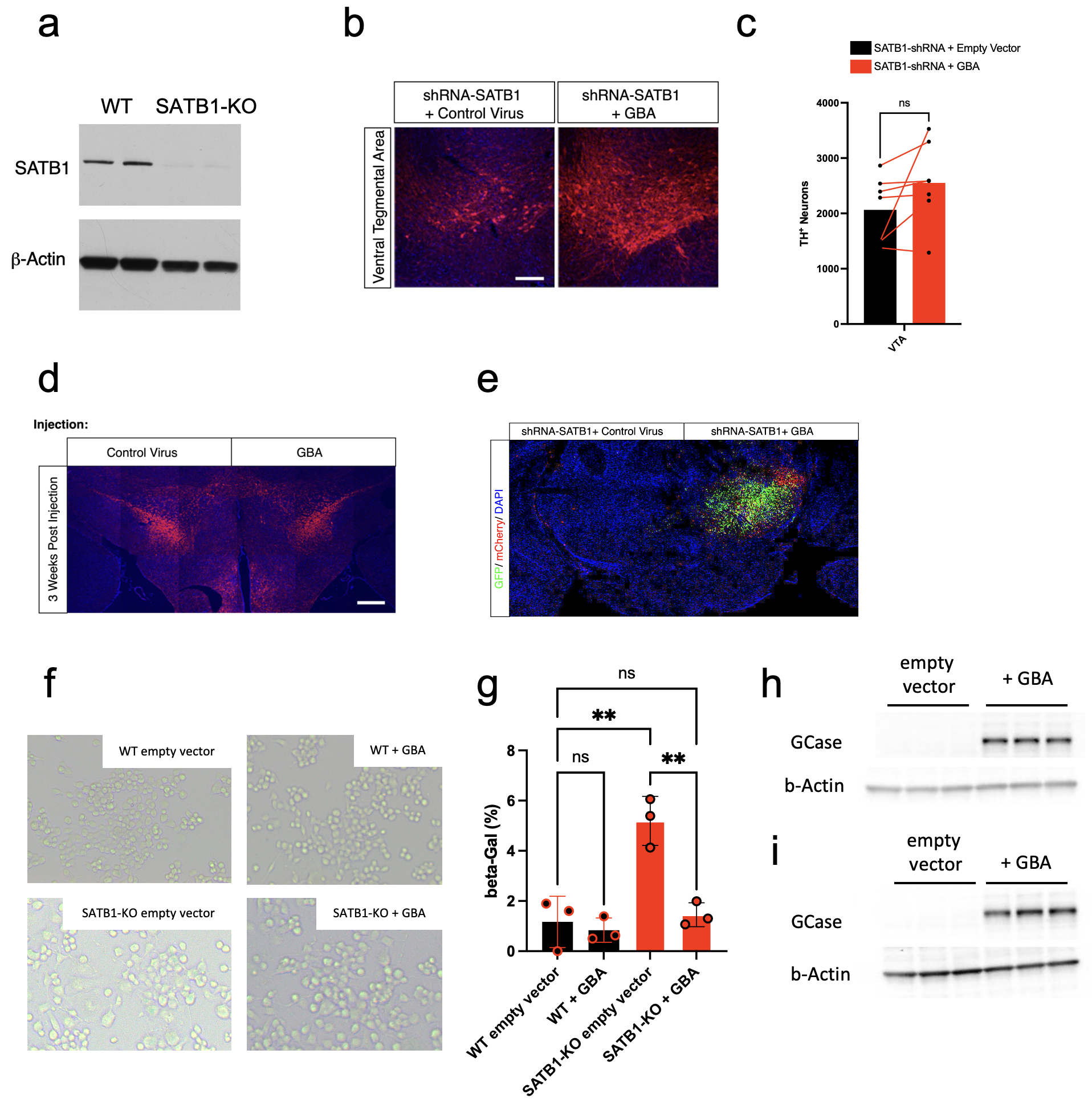
*

**Figure S2. Confirmation of SATB1 knockout (KO), *in vivo* virus experiments, and GBA-mediated rescue of SATB1-KO phenotype.**

**a,** Western blot analysis confirms the elimination of SATB1 protein in N2A SATB1-KO cells. **b** and **c,** Representative images and quantification of tyrosine hydroxylase (TH) immunofluorescent staining in mice that received a stereotaxic injection with a shRNA-SATB1 virus and a control vector or a contralateral injection with a shRNA-SATB1 virus and a GBA-overexpressing virus. The ventral tegmental area (VTA) was analyzed using unbiased stereological cell counting of TH^+^ cells, showing no significant effect (n=7). **d** and **e,** Viral overexpression of GBA in mouse dopaminergic neurons did not affect TH expression and survival of TH^+^ neurons compared to the opposite midbrain injected with the empty vector control virus, observed 3 weeks following viral injection. Scale bar: 500 μm. **f,** representative images from a SA-β-Gal assay comparing WT and SATB1-KO N2A cells transfected with empty vector or GBA overexpression plasmid (N=3, n=1311, 1673, 1663, and 1672) **g**, Quantification of the senescence assay in (**f**) showing rescue of SATB1-KO-induced senescence with GBA overexpression . **h** and **i**, representative Western blots confirming GBA overexpression in WT (**h**) and SATB1-KO (**i**) N2A cells (n=3 for each). Data are presented as mean ± S.E.M. Student’s t-test was performed for c. Two-way ANOVA was performed for f. Student’s t-test was performed for b and d. ** p<0.01; ns=not significant.


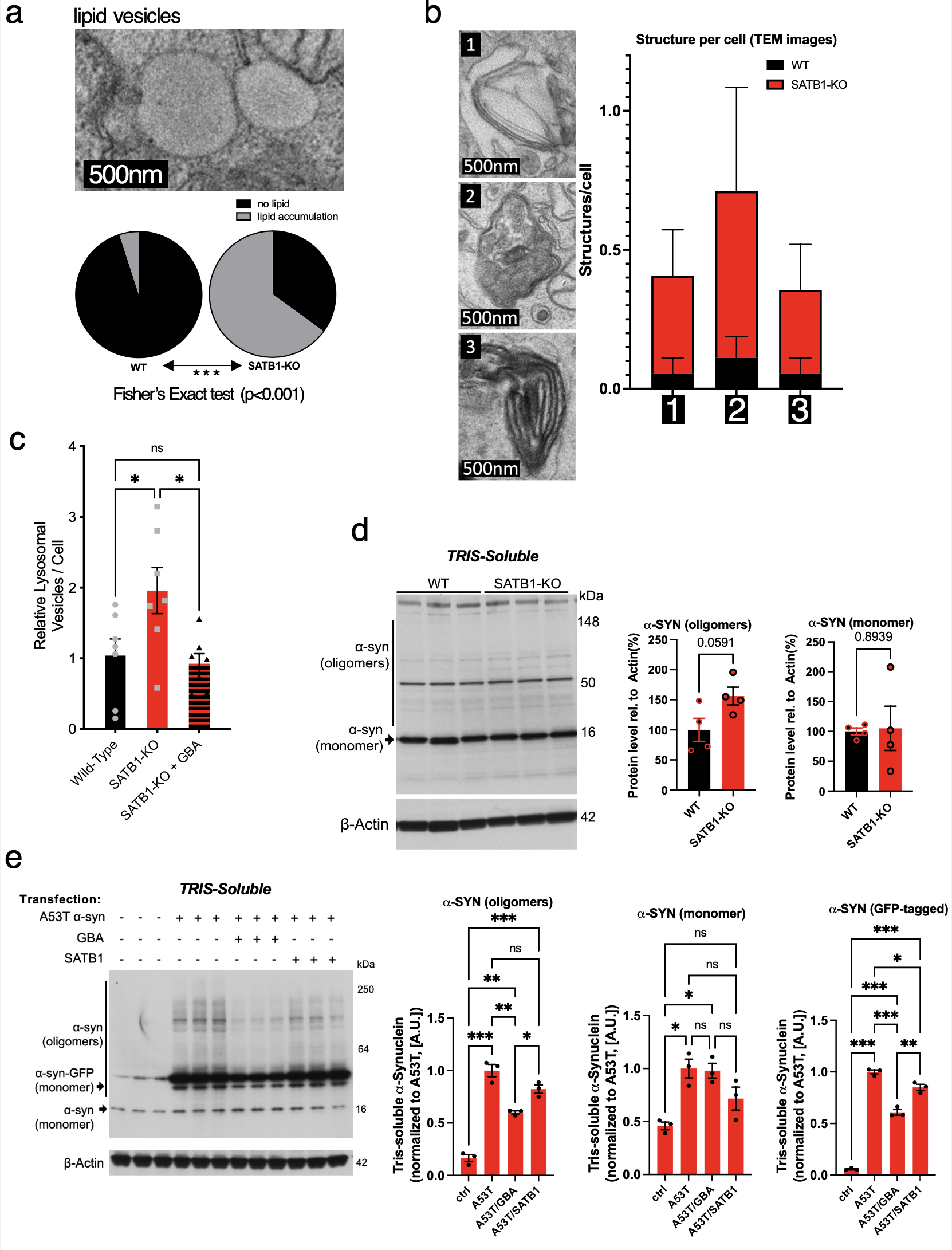


**Figure S3. Lysosomal abnormalities of SATB1-KO cells and rescue with GBA overexpression.
a,** Representative image and quantification of lipid accumulation in SATB1-KO N2A cell s (n=20) compared to wildtype (n=20). **b**, Quantification of membranous structures in WT (n=20) and SATB1-KO (n=20) N2A cells showing increase in all 3 structure types in SATB1-KO. **c,** Rescue of increased lysosomal vesicles in SATB1-KO N2A cells with GBA overexpression n=7/condition). **d**, Triton X-100 soluble α-SYN levels in N2A^WT^ (n=4) and N2A^Satb1- KO^ (n=4) cells. Representative Western blot and quantification of α-SYN monomers and oligomers are shown. **e**, Co-transfection of GBA or SATB1 along with α-SYN (A53T) reduced triton X-100 soluble α-SYN when compared to A53T transfection alone. Representative Western blot and quantifications of α-SYN monomers (with and without GFP-tag) and oligomers are shown (n=3/condition). Data are presented as mean ± S.E.M. Fisher’s exact test was performed for a. Two-way ANOVA was performed for c, and e. Unpaired Student’s t-test was performed for d. * p<0.05; ** p<0.01; *** p<0.001, ns=not significant.


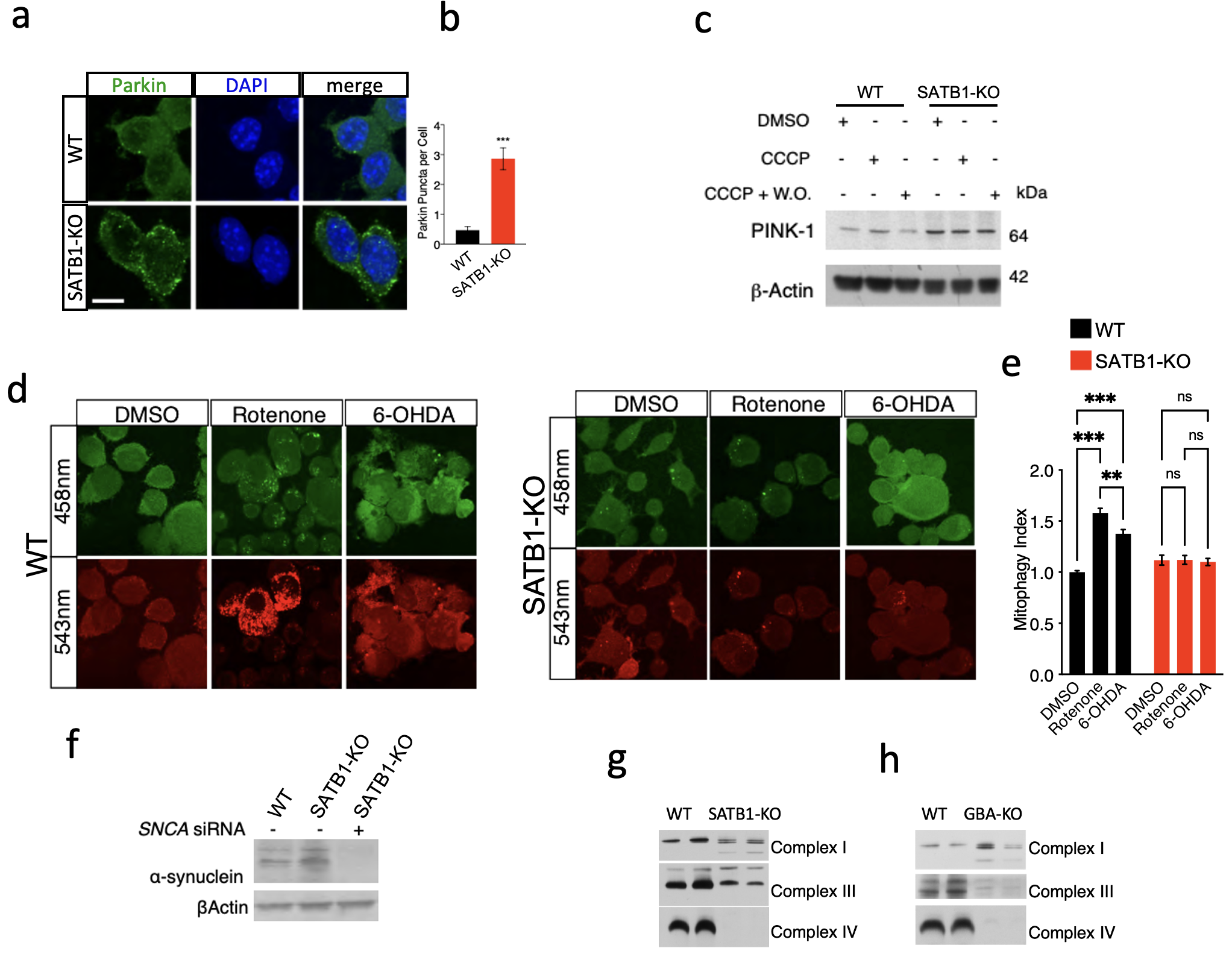


**Figure S4. SATB1 knockout (KO) leads to accumulation of dysfunctional mitochondria and altered mitophagy index.**

**a, b**, Immunofluorescent staining using anti-Parkin antibodies revealed a significant increase in Parkin-labeled puncta in N2A^SATB1-KO^ cells compared to controls. **c**, Immunoblotting analysis of PINK1 protein levels after treatment with DMSO or CCCP for 3.5 hours or CCCP followed by a 0.5-hour washout with serum-free media. PINK1 protein levels increased with CCCP treatment and returned to baseline after washout in wild-type (WT) N2A cells. However, N2A^SATB1-KO^ cells showed elevated basal levels of PINK1 protein, which remained unchanged following treatment (n=4). **d, e**, Functional analysis of mitophagy in WT and SATB1-KO N2A cells transfected with mt-mKeima plasmid and treated with rotenone or 6-OHDA. Total mitochondrial fluorescence (ex.: 458 nm) is shown in green, and mitophagy (ex.: 543) is shown in red. The mt-mKeima quantification was performed with at least 100 cells analyzed per condition. The ‘mitophagy index’ was calculated as the ratio of fluorescence intensity emitted from the two excitation peaks: 543 nm divided by 458 nm, as previously described (Goiran et al., 2022). **f,** Western blot confirmation of α-SYN knockdown in N2A cells. **g, h** Native gel electrophoresis showing loss of oxidative phosphorylation complex integrity in both SATB1-KO and GBA-KO cells. Data are presented as mean ± S.E.M. Student’s t-test was performed for b. Two-way ANOVA was performed for e. ** p<0.01 , *** p<0.001, ns=not significant.


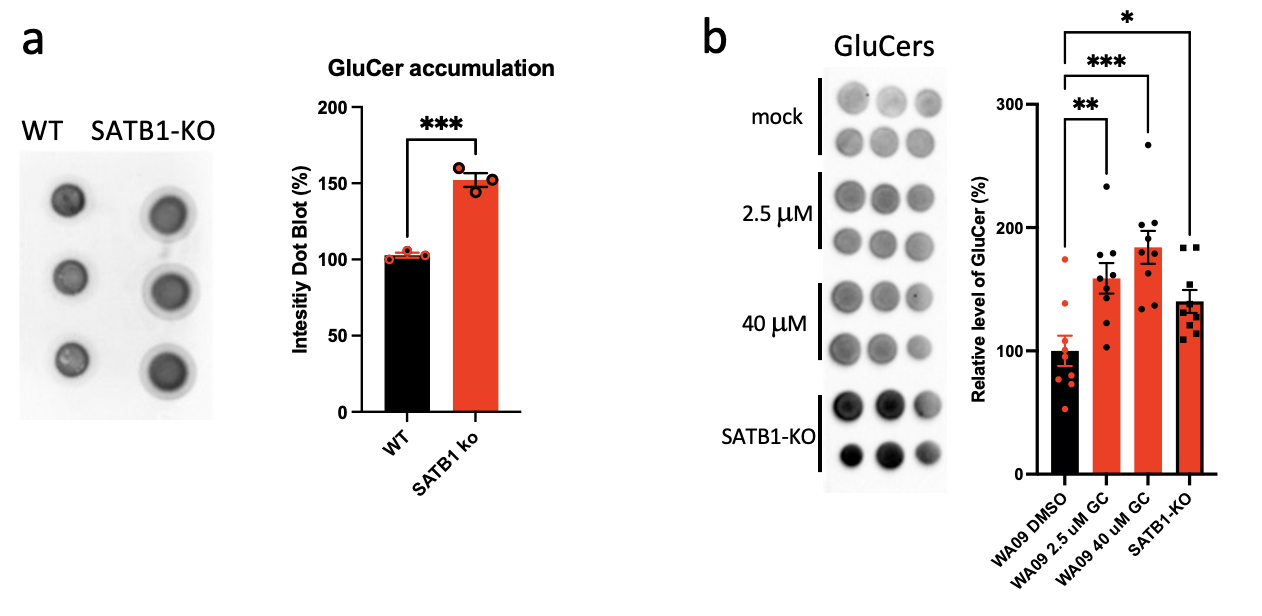


**Figure S5. Dot blot analysis of GluCer in the N2A cell line and lipid-treated dopaminergic (DA) neurons.**

**a**, Dot blot analysis in the N2A Satb1-KO cell line showing increased levels of GluCer (n=3, n=3). **b,** Dot blot analysis of GluCer in mature wild-type (WT; WA09) human DA neurons treated with DMSO control (n=9), 2.5 μM GluCer (n=9), or 40 μM GluCer (n=9), and SATB1-KO DA neurons (n=9) showing similar lipid accumulation in lipid-treated and SATB1-KO DA neurons. Data are presented as mean ± S.E.M. Student’s t-test was performed for a. Two-way ANOVA was performed for b. * p<0.05; ** p<0.01 , *** p<0.001.


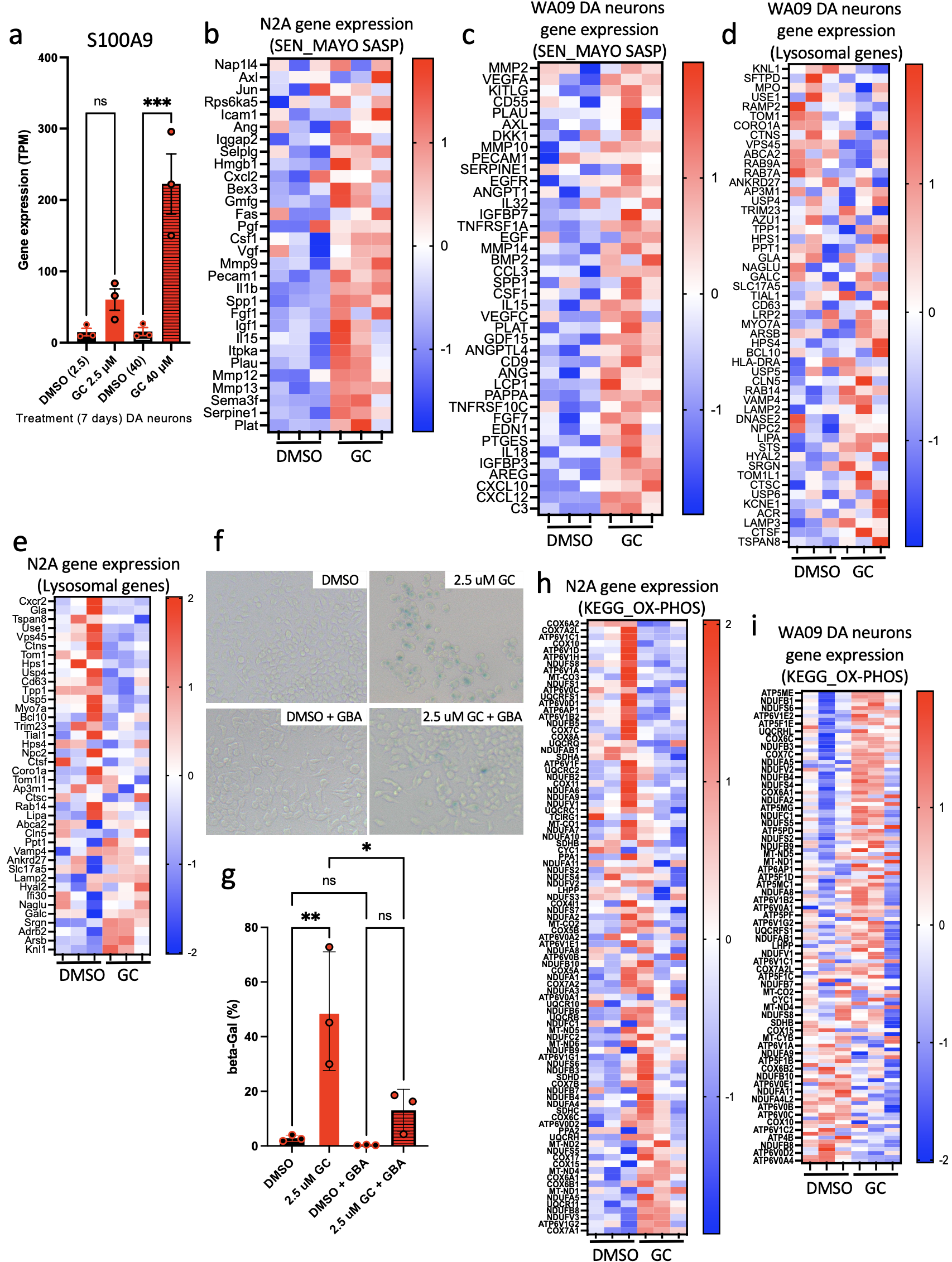


**Figure S6. RNA expression analysis and GBA-mediated rescue of GluCer treated cells.**

**a**, RNA-seq analysis of WT human DA neurons treated with DMSO (n=3), 2.5 μM (n=3), or 40 μM (n=3) GluCer revealed increased S100A9 expression following a 7-day lipid treatment. **b** and **c,** heatmaps of RNA-seq analysis of GluCer-treated N2A cells (**b**) and dopaminergic neurons (**c**) showed upregulation of various SASP factors (standardized differentially expressed genes (DEGs)) from the published SenMayo list following lipid treatment (n=3, each). **d** and **e,** RNA-seq analysis of GluCer-treated dopaminergic neurons (**d**) and N2A cells (**e**) showed dysregulation of a number of lysosomal DEGs (standardized) with lipid treatment (n=3, each) **f,** representative images from a SA-β-Gal assay comparing WT N2A cells transfected with empty vector or GBA overexpression plasmid prior to 7-day treatment with DMSO or 2.5 μM GluCer **g**, Quantification of the senescence assay in (**f**) showing rescue of GluCer-induced senescence with GBA overexpression (N=3, DMSO n=1408, DMSO + GBA n=515, 2.5 μM GluCer n=1146, and 2.5 μM GluCer + GBA n=877). **h** and **i,** heatmaps of RNA-seq analysis of GluCer-treated N2A cells (**h**) and dopaminergic neurons (**i**) showed dysregulation of various mitochondrial genes (standardized differentially expressed genes (DEGs)) from the published *KEGG_Oxidative_Phosphorylation* list following lipid treatment (n=3, each). Data are presented as mean ± S.E.M. Two-way ANOVA was performed for a and g. * p<0.05; ** p<0.01 , *** p<0.001, ns=not significant.
